# Clinical and neuroimaging association between neuropsychiatric symptoms and nutritional status across the Alzheimer's disease continuum: a longitudinal cohort study

**DOI:** 10.1016/j.jnha.2024.100182

**Published:** 2024-02-09

**Authors:** Jiwei Jiang, Anxin Wang, Hanping Shi, Shirui Jiang, Wenyi Li, Tianlin Jiang, Linlin Wang, Xiaoli Zhang, Mengfan Sun, Min Zhao, Xinying Zou, Jun Xu

**Affiliations:** a Department of Neurology, Beijing Tiantan Hospital, Capital Medical University, Beijing, 100070, China; b China National Clinical Research Center for Neurological Diseases, Beijing, 100070, China; c Department of Gastrointestinal Surgery, Beijing Shijitan Hospital, Capital Medical University, Beijing, 100038, China; d Department of Clinical Nutrition, Beijing Shijitan Hospital, Capital Medical University, Beijing, 100038, China; e Beijing International Science and Technology Cooperation Base for Cancer Metabolism and Nutrition, Beijing, 100038, China

**Keywords:** Alzheimer’s disease, Neuropsychiatric symptoms, Malnutrition, Affective symptoms, Feeding and eating disorders, Putamen

## Abstract

**Objectives:**

To investigate the association between neuropsychiatric symptoms (NPS) and nutritional status, and explore their shared regulatory brain regions on the Alzheimer’s disease (AD) continuum.

**Design:**

A longitudinal, observational cohort study.

**Setting:**

Data were collected from the Chinese Imaging, Biomarkers, and Lifestyle study between June 1, 2021 and December 31, 2022.

**Participants:**

Overall, 432 patients on the AD continuum, including amnestic mild cognitive impairment and AD dementia, were assessed at baseline, and only 165 patients completed the (10.37 ± 6.08) months’ follow-up.

**Measurements:**

The Mini-Nutritional Assessment (MNA) and Neuropsychiatric Inventory (NPI) were used to evaluate nutritional status and NPS, respectively. The corrected cerebral blood flow (cCBF) measured by pseudo-continuous arterial spin labeling of the dietary nutrition-related brain regions was analyzed. The association between the NPS at baseline and subsequent change in nutritional status and the association between the changes in the severity of NPS and nutritional status were examined using generalized linear mixed models.

**Results:**

Increased cCBF in the left putamen was associated with malnutrition, general NPS, affective symptoms, and hyperactivity (*P* < 0.05). The presence of general NPS (β = −1.317, *P* = 0.003), affective symptoms (β = −1.887, *P* < 0.001), and appetite/eating disorders (β = −1.714, *P* < 0.001) at baseline were associated with a decline in the MNA scores during follow-up. The higher scores of general NPI (β = −0.048), affective symptoms (β = −0.181), and appetite/eating disorders (β = −0.416; all *P* < 0.001) were longitudinally associated with lower MNA scores after adjusting for confounding factors.

**Conclusions:**

We found that baseline NPS were predictors of a decline in nutritional status on the AD continuum. The worse the severity of affective symptoms and appetite/eating disorders, the poorer the nutritional status. Furthermore, abnormal perfusion of the putamen may regulate the association between malnutrition and NPS, which suggests their potentially common neural regulatory basis.

## 1 Introduction

Accumulating evidence has indicated that maintaining a healthy lifestyle, which includes eating a balanced diet and ensuring optimal nutritional status, is essential for delaying memory decline and improving brain health [[1], [2], [3]]. A recent review identified a vicious cycle between clinical presentation and malnutrition in patients with Alzheimer’s disease (AD) [4]. An observational study found that the risk of malnutrition increased during AD progression and was positively associated with cognitive impairment [5]. A longitudinal study demonstrated that poorer nutritional status was associated with a higher risk of clinical progression to AD dementia [6]. However, previous studies have largely focused on the association between cognition and nutrition [7], neglecting the relationship between neuropsychiatric symptoms (NPS), an important and prevalent group of non-cognitive symptom clusters on the AD continuum, and nutrition.

NPS comprise a group of highly heterogeneous sub-symptoms, including hallucinations, agitation, depression, and anxiety [8]. They are generally associated with poorer cognition, quality of life, and faster disease progression [9]. However, the causal relationship between NPS and nutritional status on the AD continuum remains to be elucidated. A cross-sectional study found that nutritional problems were associated with individual NPS in older women with mild cognitive impairment (MCI) and early-stage AD [10]. A longitudinal study demonstrated that worse nutritional status was associated with more severe NPS, including psychosis, depression, and apathy, in patients with dementia [11]. Despite accumulating evidence, previous studies have either included only women as the study population or various types of dementia. Moreover, these studies have merely focused on the effect of nutritional status on the changes in NPS. Therefore, it is difficult to ascertain whether malnutrition is a consequence of AD-related NPS in the abovementioned vicious cycle.

In addition, the potential mechanism that affects both NPS and nutritional status is yet to be elucidated. The possibility of a shared regulatory brain region that can explain the association between NPS and nutrition needs to be explored. Thus, this study aimed to investigate the baseline and long-term association between NPS and nutritional status in patients on the AD continuum, and explored the potential shared brain regions that regulate both NPS and nutritional status, providing evidence on the vicious cycle between malnutrition and AD continuum.

## 2 Methods

### 2.1 Study design and population

**Fig. 1:**
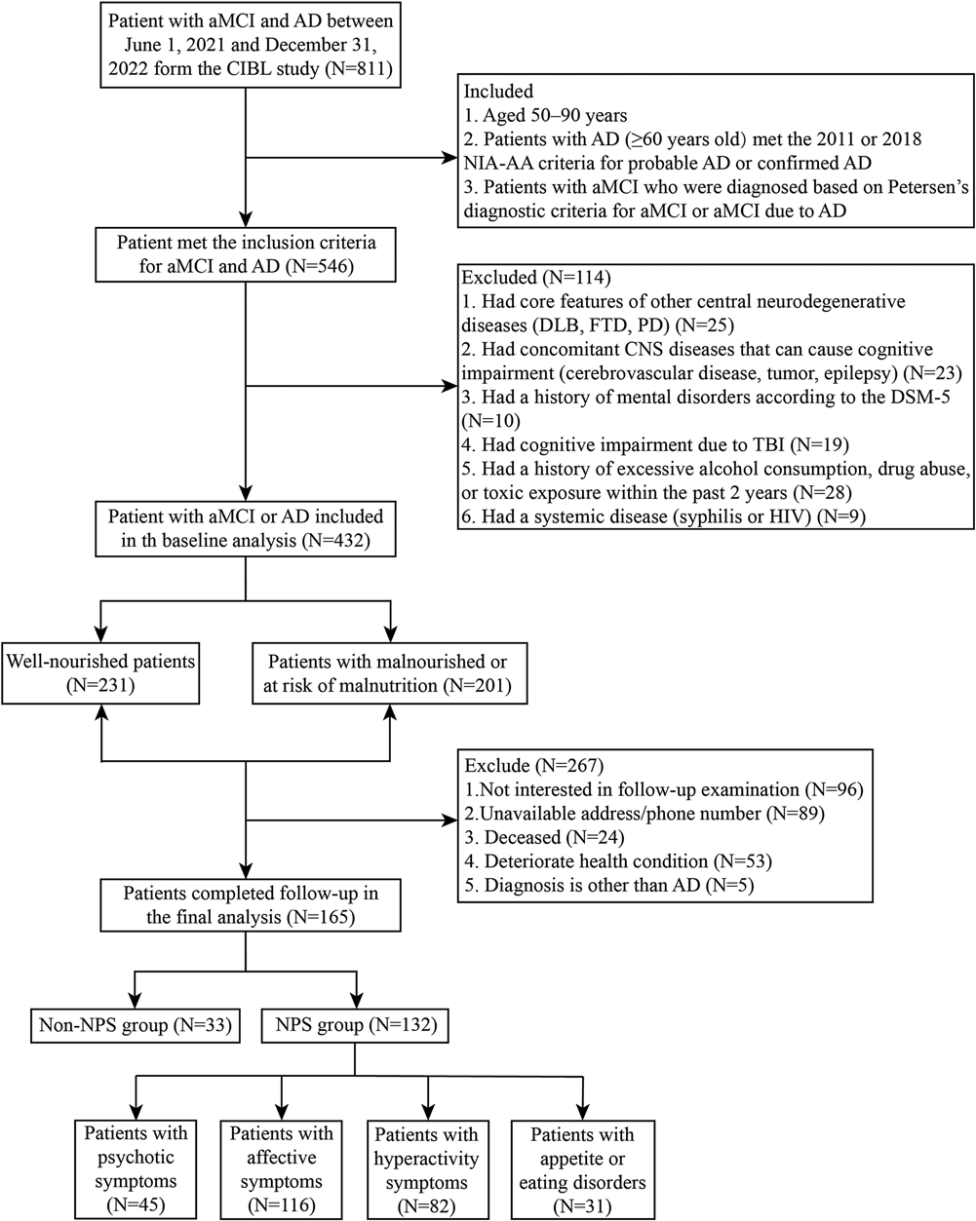
Detailed flowchart of the recruitment and analysis processes. aMCI, amnestic mild cognitive impairment; AD, Alzheimer’s disease; CIBL, the Chinese Imaging, Biomarkers, and Lifestyle study; NIA-AA, National Institute on Aging Alzheimer’s Association; MoCA, the Montreal Cognitive Assessment; DLB, dementia with Lewy bodies; FTD, frontotemporal dementia; PD, Parkinson’s disease; DSM-5, Diagnostic and Statistical Manual of Mental Disorders-5; TBI, traumatic brain injury; HIV, human immunodeficiency virus; and NPS, neuropsychiatric symptom.

This study included patients on the AD continuum at baseline, including amnestic MCI (aMCI) and AD dementia, enrolled from the Chinese Imaging, Biomarkers, and Lifestyle (CIBL) study between June 1, 2021 and December 31, 2022. The CIBL study, as previously described [12], was approved by the Institutional Review Board of Capital Medical University, Beijing Tiantan Hospital (approval number: KY-2021-028-01) and registered on chictr.org.cn (number: ChiCTR2100049131). All the participants or their caregivers (if necessary) provided written informed consent. The inclusion criteria were as follows: patients aged 50–90 years; patients with AD (age of onset >60 years) who met the 2011 or 2018 National Institute on Aging Alzheimer’s Association (NIA-AA) workgroup diagnostic criteria for probable AD or confirmed AD [13,14]; and patients with aMCI who were diagnosed based on MCI due to AD based according to the 2018 NIA-AA diagnostic criteria [14] or Petersen’s diagnostic criteria for aMCI [15]. Trained research coordinators conducted in-person interviews of patients with aMCI and AD dementia at 6-month intervals. The assessment contents were identical to those at baseline. The information was obtained directly from the participants by telephone calls without face-to-face follow-ups. Fig. 1 depicts a detailed flowchart of the recruitment and analysis processes.

### 2.2 Data collection

#### 2.2.1 Clinical data collection

Demographic data, including age, sex, years of education, marital status, clinical diagnosis; medical history, including hypertension, diabetes mellitus, cerebrovascular disease, coronary heart disease, and dyslipidemia; and apolipoprotein E-type epsilon 4 (*APOE*ε4) status were collected at baseline.

#### 2.2.2 Nutritional assessment

The nutritional status was evaluated using the Mini-Nutritional Assessment (MNA) [16], which comprises simple measurements and brief questions, including anthropometric measurements, a global assessment, a dietary questionnaire, and a subjective assessment. The total MNA score was used to distinguish between well-nourished patients (≥ 24), patients at risk of malnutrition (17–23.5), and malnourished patients (<17). The higher the MNA score, the better the nutritional status.

#### 2.2.3 NPS and sub-symptoms assessment

NPS and relevant sub-symptoms were evaluated using the Neuropsychiatric Inventory (NPI), including the following 12 different behavioral and neuropsychiatric domains: delusion, hallucination, agitation, depression, anxiety, euphoria, apathy, disinhibition, irritability, aberrant motor activity, sleep/nighttime behavior, and appetite/eating disturbances [17]. The total NPI severity score is calculated by summing the 12 severity sub-scores (severity score × frequency score in each reported symptom). Higher NPI scores correspond with worse NPS or relevant sub-symptoms. Considering the highly correlated and contingent co-occurrence of numerous individual symptom clusters, we divided the 12 statistically significant individual symptoms into three main symptom clusters as previously described [18]: psychotic symptoms (hallucinations and delusions), affective symptoms (depression, anxiety, and apathy), and hyperactivity (agitation, disinhibition, irritability, and aberrant motor behavior). Appetite/eating disorders, euphoria, and sleep and nighttime behavior were included as individual sub-symptoms.

#### 2.2.4 Other comprehensive neuropsychological tests

Global objective cognition was assessed using the Chinese version of the Mini-Mental State Examination (MMSE) [19] and the Beijing version of the Montreal Cognitive Assessment (MoCA) [20]. The Activities of Daily Living (ADL) Questionnaire was completed by the caregivers to evaluate patients’ ability to perform daily activities. The Pittsburgh Sleep Quality Index (PSQI) was used to asses patients’ sleep quality [21]. The Caregiver Burden Inventory (CBI) was used to quantify the impact of caregivers’ burden [22].

### 2.3 Neuroimaging data acquisition and analysis

A 3.0 T magnetic resonance scanner (SIGNA Premier; GE Healthcare, Milwaukee, WI, USA) was used for image acquisition of 7-delay pseudo-continuous arterial spin labeling (pCASL) in all participants. The parameters were as follows: repetition time = 9315.0 ms, echo time = 11.2 ms, field of view = 220 mm × 220 mm, acquisition matrix = 512 × 512, 48 axial slices, and thickness = 3.0 mm. The post-labeling delays were 1.000, 1.361, 1.739, 2.141, 2.577, 3.067, and 3.658 s. The data was independently processed using CereFlow software 1.0 (Anying Technology Beijing Co., Ltd., Beijing, China) by two radiologists. The brain regions of interest (ROIs) in this study were the regions involved in both the homeostatic regulation of appetite and cognitive control of eating, which is consistent with previous reports [[23], [24], [25]]. Finally, the regional corrected cerebral blood flow (cCBF) and arterial transit time (ATT) values of 19 ROIs, including the bilateral insula, amygdala, caudate nucleus, putamen, nucleus accumbens (NAc), ventral tegmental area (VTA), locus coeruleus (LC), anterior cingulate cortex (ACC), and hypothalamus, as well as the dorsal raphe nucleus, were extracted according to the third edition of the automated anatomical atlas (AAL3) [26].

### 2.4 Statistical analysis

All statistical analyses were performed using SAS Version 9.4 software (SAS Institute, Inc., Cary, NC, USA) and SPSS version 26.0 (SPSS Inc., Chicago, IL, USA). Categorical variables are presented as proportions and were compared using the χ² test, whereas continuous variables are presented as mean ± standard deviation (SD) or median interquartile range. Comparisons were performed using independent *t*-tests for data with normal distribution or the Mann–Whitney U test for skewed data. Multivariate logistic stepwise regression analysis was performed to determine independent clinical factors contributing to malnutrition or the risk of malnutrition and to identify the independent brain regions that affect both malnutrition and NPS after adjusting for confounding factors.

The generalized estimating equation was used to identify the association between the presence of NPS and four sub-symptom clusters at baseline and the changes in MNA scores in patients after the follow-up period without adjustment. The generalized linear mixed-effects models were used to identify the longitudinal association between the presence or severity of NPS and MNA scores in patients with aMCI or AD during the follow-up period, adjusting for age, sex, history of diabetes mellitus, ADL scores, and follow-up duration.

## 3 Results

### 3.1 Baseline clinical characteristics

We analyzed 432 patients with aMCI (232, 53.70%) or AD dementia (200, 46.30%) at baseline; of whom, 231 (53.47%) were well-nourished, and 201 (46.53%) were malnourished or at risk of malnutrition. Table 1 presents the baseline characteristics of patients on the AD continuum according to different nutritional statuses. Malnourished individuals or those at risk of malnutrition were older, predominantly female, and had a history of diabetes mellitus, compared with well-nourished patients (all *P* < 0.05). Patients with or at risk of malnutrition had higher NPI scores and a higher frequency of NPS, psychotic symptoms, affective symptoms, hyperactivity, and appetite/eating disorders (all *P* < 0.001), compared with well-nourished patients. Patients with malnutrition or at risk of malnutrition had lower MMSE, MoCA, and MNA scores (all *P* < 0.001) but higher ADL and CBI scores (all *P* < 0.001), compared with well-nourished patients. No significant differences were observed in marital status, education, *APOE*ε4 status, the prevalence of comorbidities (hypertension, dyslipidemia, cerebrovascular disease, and coronary heart disease), the prevalence of euphoria and sleep/nighttime behavior, and the PSQI scores between the two groups (all *P* > 0.05).

**Table 1:** Baseline characteristics of the patients with aMCI and AD by different nutritional statuses.

Variable	Overall (N = 432)	Well-nourished (N = 231)	At risk of malnutrition/malnourished (N = 201)	t/χ^2^/Z	*P* value
Demographics					
Age [years, median (IQR)]	67.11 ± 8.37	65.76 ± 7.63	68.66 ± 8.91	−2.899	<0.001
Sex (female, %)	264 (61.11)	130 (56.28)	134 (66.67)	4.882	0.027
Education [years, median (IQR)]	11.00 (8.13, 12.00)	11.00 (9.00, 13.00)	11.00 (8.00, 12.00)	−1.811	0.070
Marital status (married, %)	371 (85.88)	196 (84.85)	175 (87.06)	0.435	0.509
*APOE∑4* carrier (yes, %)	143 (33.10)	70 (30.30)	73 (36.32)	1.756	0.185
Medical Histories					
Hypertension (yes, %)	192 (44.44)	101 (43.72)	91 (45.27)	0.105	0.746
Diabetes mellitus (yes, %)	82 (18.98)	32 (13.85)	50 (24.88)	8.492	0.004
Cerebrovascular disease (yes, %)	79 (18.29)	35 (15.15)	44 (21.89)	3.267	0.071
Coronary heart disease (yes, %)	86 (19.91)	47 (20.35)	39 (19.40)	0.060	0.807
Dyslipidemia (yes, %)	185 (42.82)	104 (45.02)	81 (40.30)	0.979	0.322
NPS and sub-symptoms					
NPI (score, median [IQR])	4.50 (1.00, 14.00)	2.00 (0.00, 6.00)	10.00 (4.00, 22.00)	−9.213	<0.001
NPSs (yes, %)	326 (75.46)	145 (62.77)	181 (90.05)	43.195	<0.001
Psychotic symptoms (yes, %)	103 (23.84)	34 (14.72)	69 (34.33)	22.762	<0.001
Affective symptoms (yes, %)	275 (63.66	112 (48.48)	163 (81.10)	49.403	<0.001
Hyperactivity (yes, %)	196 (45.37)	81 (35.06)	115 (57.21)	22.692	<0.001
Appetite or eating disorders (yes, %)	61 (14.12)	11 (4.76)	50 (24.88)	26.045	<0.001
Euphoria (yes, %)	30 (6.94)	13 (5.63)	17 (8.46)	1.332	0.248
Sleep and nighttime behavior (yes, %)	123 (28.47)	58 (25.11)	65 (32.33)	2.759	0.097
Neuropsychological battery					
MMSE (score, median [IQR])	23.00 (16.00, 26.00)	24.00 (20.00, 27.00)	20.00 (11.00, 25.00)	−6.965	<0.001
MoCA (score, median [IQR])	18.00 (11.00, 22.00)	19.00 (14.00, 23.00)	14.00 (6.00, 20.00)	−6.876	<0.001
MNA (score, median [IQR])	24.00 (21.50, 26.00)	25.75 (24.50, 27.00)	21.00 (19.00, 22.50)	−17.958	<0.001
ADL (score, median [IQR])	21.00 (20.00, 29.00)	20.00 (20.00, 22.00)	25.00 (20.00, 37.00)	−7.915	<0.001
PSQI (score, median [IQR])	5.00 (2.00, 8.00)	5.00 (2.00, 8.00)	5.00 (2.00, 9.00)	−0.995	0.320
CBI (score, median [IQR])	3.00 (0.00, 21.00)	0.00 (0.00, 9.75)	10.00 (0.00, 28.00)	−6.646	<0.001

χ^2^ tests, independent *t*-tests, and Mann–Whitney U test. Data are shown as mean ± standard deviation, median (interquartile range), or n (%).

Abbreviation: aMCI, amnestic mild cognitive impairment; AD, Alzheimer’s disease; *APOE∑4*, the apolipoprotein E type epsilon 4; NPI, Neuropsychiatric Inventory; NPS, neuropsychiatric symptoms; MMSE, Mini-Mental State Examination; MoCA, Montreal Cognitive Assessment; MNA, Mini-nutritional Assessment; ADL, Activities of Daily Living; PSQI, Pittsburgh Sleep Quality Index; and CBI, caregiver burden inventory.

### 3.2 Multivariate logistic regression analyses in clinical parameters at baseline

**Fig. 2:**
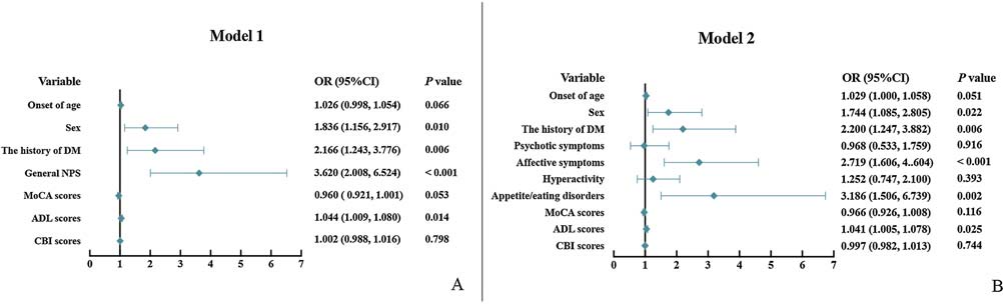
Forest plot based on multivariate logistic regression of baseline clinical characteristics. Abbreviations: NPS, neuropsychiatric symptoms; DM, diabetes mellitus; MoCA, the Montreal Cognitive Assessment; ADL, Activities of daily living; and CBI, caregiver burden inventory.

Considering the collinearity, two models based on multivariate logistic stepwise regression were used to identify the factor independently associated with malnutrition. The first model included seven factors (age, sex, history of diabetes mellitus, presence of NPS, MoCA, ADL, and CBI scores) that contributed to malnutrition or risk of malnutrition that were statistically significant in the univariate analysis at baseline. The second model included ten factors (age, sex, history of diabetes mellitus, the presence of psychotic symptoms, affective symptoms, hyperactivity, appetite/eating disorders, MoCA, ADL, and CBI scores). The findings are presented in Fig. 2.

In the first model, female sex [odds ratio (OR) = 1.836, 95% confidence interval (CI): 1.156–2.917, *P* =  0.010], history of diabetes mellitus (OR = 2.166, 95% CI: 1.243–3.776, *P* =  0.006), presence of NPS (OR = 3.620, 95% CI: 1.210–4.569, *P* =  0.012), and higher ADL scores (OR = 1.044, 95% CI: 1.009–1.080, *P* =  0.014) were significant independent risk factors for malnutrition in patients with aMCI and AD (Fig. 2A). The second model revealed that female sex (OR = 1.744, 95% CI: 1.085–2.850, *P* =  0.022), history of diabetes mellitus (OR = 2.200, 95% CI: 1.247–3.882, *P* =  0.006), presence of affective symptoms (OR = 2.719, 95% CI: 1.606–4.604, *P* <  0.001), and appetite/eating disorders (OR = 3.186, 95% CI: 1.506–6.739, *P* =  0.002), and higher ADL scores (OR = 1.041, 95% CI: 1.005–1.078, *P* =  0.023) were significant independent risk factors for malnutrition in patients with aMCI and AD (Fig. 2B).

### 3.3 ROIs associated with nutritional status and specific NPS at baseline

**Fig. 3:**
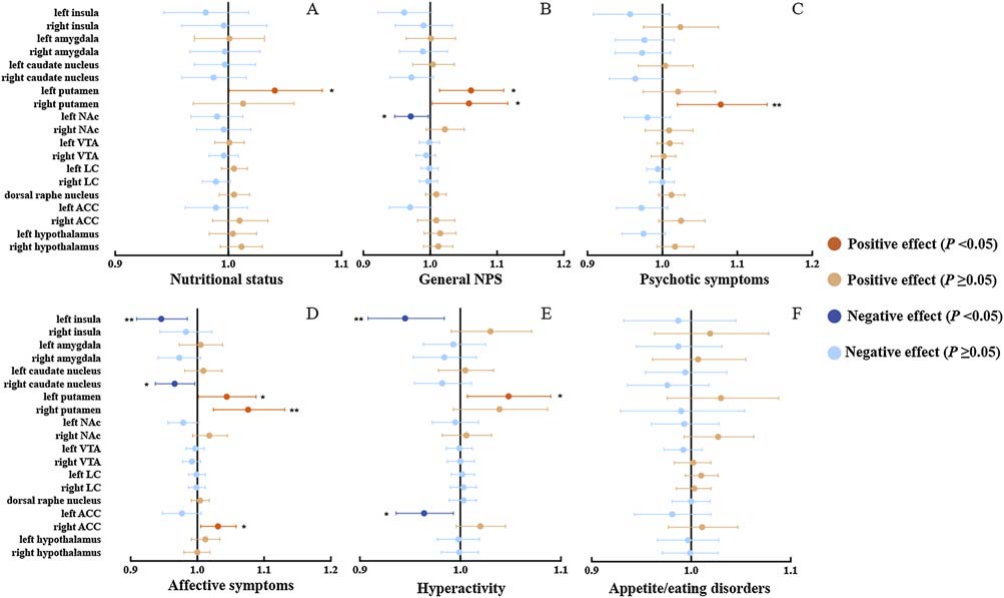
Forest plot of the association between cCBF values of 19 ROIs and nutritional status or NPS and its sub-symptom clusters at baseline after adjustment for age and sex. Abbreviation: ROIs, regions of interest; NPS, neuropsychiatric symptoms; NAc, nucleus accumbens; VTA, ventral tegmental area; LC, locus coeruleus; ACC, anterior cingulate cortex

Fig. 3 demonstrates the association of 19 ROIs with nutritional status and general or specific NPS at baseline. After adjustment for age and sex, the increased cCBF of the left putamen was independently associated with malnutrition (OR = 1.041, 95% CI: 1.001–1.083, *P* =  0.043). The increased cCBF of the left and right putamen were independently associated with general NPS (OR = 1.061, 95% CI: 1.014–1.110, *P* =  0.011; OR = 1.058, 95% CI: 1.003–1.116, *P* =  0.037), while the decreased cCBF of the left NAc was independently associated with general NPS (OR = 0.971, 95% CI: 0.947–0.997, *P* =  0.027). The increased cCBF of the right putamen was independently associated with psychotic symptoms (OR = 1.078, 95% CI: 1.020–1.140, *P* =  0.008). The decreased cCBF of the left insula and right caudate nucleus were independently associated with affective symptoms (OR = 0.946, 95% CI: 0.909–0.985, *P* =  0.006; OR = 0.966, 95% CI: 0.937–0.996, *P* =  0.027), while the increased cCBF of the left putamen, right putamen, and right ACC were independently associated with affective symptoms (OR = 1.044, 95% CI: 1.002–1.088, *P* =  0.040; OR = 1.076, 95% CI: 1.024–1.131, *P* =  0.004; OR = 1.031, 95% CI: 1.005–1.058, *P* =  0.018). The decreased cCBF of the left insula and ACC were independently associated with hyperactivity (OR = 0.945, 95% CI: 0.908–0.984, *P* =  0.005; OR = 0.964, 95% CI: 0.936–0.993, *P* =  0.014), while the increased cCBF of the left putamen was independently associated with hyperactivity (OR = 1.048, 95% CI: 1.007–1.090, *P* =  0.021). Detailed data can be found in Supplementary Table S1.

### 3.4 Longitudinal association between NPS and MNA scores during the follow-up period

Overall, 165 patients (38.19%) completed the follow-up, with an average of 10.37 ± 6.08 months in this study. No significant differences were observed in the baseline clinical characteristics between patients who completed follow-up and those lost to follow-up (all *P* > 0.05, Supplementary Table S2).

The association between the presence of NPS and sub-symptom clusters at baseline with changes in the MNA score during the follow-up period is presented in Table 2. Univariate analysis revealed that the presence of NPS (β = -2.494, *P* <  0.001), psychotic symptoms (β = -1.620, *P* =  0.030), affective symptoms (β = -2.821, *P* <  0.001), hyperactivity (β = -1.593, *P* =  0.001), and appetite/eating disorders (β = -2.830, *P* <  0.001) at baseline was significantly associated with a decline in MNA score over the follow-up period. Multivariate regression analysis revealed that the presence of NPS (β = -1.317, *P* =  0.003), affective symptoms (β = -1.887, *P* <  0.001), and appetite/eating disorders (β = -1.714, *P* <  0.001) at baseline was also significantly associated with a decline in the MNA score over the follow-up period, after adjustment for age, sex, history of diabetes mellitus, ADL scores, and follow-up duration.

**Table 2:** Association of the presence of general NPS and sub-symptom clusters at baseline with changes in the MNA score during the follow-up period.

Variables	Baseline MNA scores	Follow-up MNA scores	Univariate analysis (GEE)			Multivariate analysis (GLMM)		
			β (95%CI)	Z value	*P* value	β (95%CI)	F value	P value
General NPS								
Yes (N = 132)	24.00 (20.50, 26.38)	23.00 (20.50, 25.00)	−2.494 (−3.360, −1.629)	−5.65	<0.001	−1.317 (−2.183, −0.451)	8.95	0.003
No (N = 33)	26.00 (24.50, 27.00)	25.00 (23.75, 26.75)						
Psychotic symptoms								
Yes (N = 45)	22.50 (20.00, 25.00)	21.50 (19.50, 23.00)	−1.620 (−2.690, −0.550)	−2.97	0.030	−0.106 (−0.951, 0.739)	0.06	0.806
No (N = 125)	24.50 (22.00, 26.50)	24.00 (22.00, 26.00)						
Affective symptoms								
Yes (N = 116)	23.00 (20.00, 25.00)	22.75 (20.00, 24.50)	−2.821 (-3.645, -1.992)	−6.71	<0.001	−1.887 (−2.626, −1.148)	25.23	<0.001
No (N = 49)	26.00 (24.50, 27.00)	25.00 (23.50, 26.75)						
Hyperactivity								
Yes (N = 82)	23.75 (20.50, 25.13)	22.50 (20.00, 24.50)	−1.593 (−2.535, −0.651)	−3.31	0.001	−0.525 (−1.230, 0.181)	2.14	0.145
No (N = 83)	25.00 (22.50, 27.00)	24.50 (23.00, 26.00)						
Appetite/eating disorders								
Yes (N = 31)	21.50 (19.00, 24.00)	21.50 (18.50, 23.00)	−2.830 (−4.045, −1.616)	−4.57	<0.001	−1.714 (−2.591, −0.839)	14.78	<0.001
No (N = 134)	24.50 (22.38, 26.63)	24.00 (21.50, 26.00)						

Adjusted for age, sex, the medical history of diabetes mellitus, ADL scores, and follow-up time interval. NPS, neuropsychiatric symptom; MNA, Mini-nutritional Assessment; GEE, generalized estimating equation, GLMM, generalized linear-mixed effects model; and ADL, Activities of daily living.

### 3.5 Longitudinal association of the changes in general NPI score and sub-symptom clusters score with MNA score during follow-up

During the follow-up period, the higher NPI scores (β = −0.048, 95% CI −0.072 to −0.025, F = 16.57, *P* <  0.001), higher sub-symptom cluster scores for affective symptoms (β = −0.181, 95% CI −0.238 to −0.125, F = 39.45, *P* <  0.001), and appetite/eating disorders (β = −0.416, 95% CI −0.593 to −0.239, F = 21.44, *P* <  0.001) were significantly associated with lower MNA scores after adjusting for confounding factors. Neither the longitudinal changes in the sub-symptom cluster scores for psychotic symptoms (*P* =  0.064) nor hyperactivity (*P* =  0.489) were associated with the MNA score.

## 4 Discussion

This study found that the presence of NPS, especially affective symptoms and appetite/eating disorders at baseline, were associated with the subsequent decline in nutritional status over the follow-up period in patients on the AD continuum. Moreover, the more severe the affective symptoms or appetite/eating disorders, the poorer the nutritional status over the follow-up period. These findings highlight the longitudinal impact of NPS and their sub-symptom clusters on nutritional status on the AD continuum. Furthermore, it suggests that clinicians should actively take measures to prevent malnutrition in patients in the early stages of AD when they present with affective symptoms or appetite/eating disorders. More importantly, this is the first study to demonstrate that abnormal blood perfusion in the putamen is associated with both nutritional status and NPS, revealing that putamen may be a shared neural regulatory basis of the association between these two factors.

Our study found that affective symptoms, including depression, apathy, and anxiety, at baseline were risk predictors for malnutrition or risk of malnutrition on the AD continuum. It provides clear evidence on the previous unestablished link between nutrition and NPS in patients with AD [27,28]. Moreover, we found that more serious affective symptoms were associated with worse nutritional status as disease progression, consistent with the previous findings. For instance, a longitudinal study suggested that apathy had a strong association with weight loss in nursing home residents [29]. A retrospective study that enrolled 157 outpatients with mild-to-moderate AD demonstrated that depression was a common risk factor for pre-frailty and frailty, a special manifestation of malnutrition [30]. This suggests that clinicians should consider affective symptoms as an important etiology of declining nutritional status in patients on the AD continuum and actively take measures to prevent malnutrition in the early stages.

We postulated two possible reasons for this relationship. First, the loss of appetite and decreased food intake were core presentations of apathy, anxiety, and depression, conditions which can lead to reduced nutritional intake and energy and further affect nutritional status [31]. Second and more importantly, affective symptoms and nutrition may share common regulatory brain regions, and the impairment of these regions could affect the neurotransmission and nutrient intake and absorption [32]. This study is the first to identify that abnormal perfusion of the left putamen was independently associated with both malnutrition and affective symptoms, suggesting that the potential regulatory impact of putamen could be serve as their shared neural basis. A retrospective study also found that the higher consumption of ultra-processed foods and drinks was associated with higher depressive symptoms in participants with nutritional problems and lower volumes of the left ventral putamen [33]. An observational study found that patients with AD who have anxiety had significant gray matter volume changes in the bilateral putamen than that observed in those without [34]. A meta-analysis also found that putamen atrophy in patients with AD was associated with apathy, potentially independent of cognitive impairment and depression [35]. Accordingly, a study based on 26,466 UK Biobank participants demonstrated that higher Mediterranean-DASH Intervention for Neurodegenerative Delay diet adherence was associated with slower atrophy in the putamen, regardless of the genetic predispositions of AD [36]. A further study focused on postpartum women found that the putamen appeared to encode for visual food cue processing and nutrition selection [37]. These findings along with our study’s results provide novel insights into the shared brain area (putamen) and its ability to regulate both affective symptoms and malnutrition. However, direct evidence regarding the mechanism of putamen in the regulation of both nutrition and NPS in patients with AD is lacking. Mesolimbic dopaminergic circuit (from the midbrain to striatum) is well-known to be essential for food reward and motivational behaviors and can contribute to changes in energy balance and emotion [38]. A recent review summarized that mesolimbic dopamine involved in the striatum was associated with motivational symptoms of depression, including fatigue, apathy, and perceived loss of energy [39]. Another review suggested that aversive stressful events could negatively regulate the dopaminergic reward system, which perturbed reward sensitivity and was closely associated with chronic stress-induced depression [40]. The evidence revealed the intriguing complexity of the relationship between NPS and malnutrition and highlighted the potential neurobiological mechanisms of their regulation by putamen.

Appetite/eating disorders in patients with different severities of AD usually comprise swallowing problems, changes in appetite, food preferences, eating habits, and other oral behaviors [41]. Although it is not difficult to understand that the loss of appetite and imbalanced feeding may affect patients’ nutritional status and energy, this hypothesis is not supported by copious evidence. However, our study found that appetite/eating disorders at baseline could be considered as predictors of nutritional status decline in patients on the AD continuum. Moreover, more serious appetite/eating disorders were associated with worse nutritional status during follow-up, which emphasized the effect of appetite/eating disorders on the nutritional status trajectory of patients on the AD continuum. A recent study showed several possible relationships of malnutrition with food attitudes and preferences variables, but these associations require further exploration [42]. Another study found that individuals with AD often present with eating behaviors that could promote malnutrition, such as spilling food from the mouth, swallowing difficulties, and clamping the mouth shut [43]. A cross-sectional study conducted in 60 community-dwelling older adults with dementia found that eating difficulties and impaired mental status were closely associated with weight loss [44]. Another observational study that investigated 345 patients with mild dementia and a Clinical Dementia Rating of 0.5 also revealed that underweight patients had significantly higher scores for loss of appetite and swallowing problems than well-nourished patients [45]. Moreover, a recent review summarized that eating disorders were associated with numerous medical complications, generally due to starvation, malnutrition, and their associated physiological effects [46]. Although we did not identify a significant association between the putamen and eating disorders, a meta-analysis identified a significant hyperactivity cluster in the right putamen in participants with disordered eating than in those without [47]. A recent study also found that multivariate connectivity of the sensorimotor putamen was altered in humans with severe disordered eating behavior [48]. These findings suggest that additional energy sources and nutrition should be supplied to such patients to prevent malnutrition when appetite/eating disorders occur in patients on the AD continuum. Furthermore, treating affective and appetite/eating symptoms could serve as a potential interventional strategy in lowering the risk of malnutrition, not simply restricted to alleviating NPS itself.

This study had various strengths. It is the first to evaluate the longitudinal effect and association of NPS and the sub-symptoms with nutritional status on the AD continuum. The prospective longitudinal design allowed for valuable long-term evidence. Moreover, it is the first to reveal that the shared brain area regulating NPS and malnutrition is the putamen. However, this study also has some limitations. First, owing to the coronavirus disease 2019, some patients were lost during follow-up, resulting in a relatively small follow-up sample size. However, no significant differences were observed in the baseline clinical characteristics between those lost during the follow-up and those who completed the follow-up process. Second, we only investigated parameters at one follow-up time point, which was relatively short given the clinical course of patients on the AD continuum.

## 5 Conclusion

This study revealed that the presence of NPS, especially affective symptoms and appetite/eating disorders, at baseline, were significantly associated with the subsequent decline in nutritional status. Furthermore, long-term nutritional status declined as the affective symptoms and appetite/eating disorders became more serious. These findings strengthen the research evidence regarding the vicious cycle and causal relationship between NPS and malnutrition on the AD continuum. This study recommends clinicians to take active measures to assess and prevent malnutrition when patients present with affective symptoms or appetite/eating disorders during admission. Furthermore, this study provides novel insights into the shared brain regions (putamen) that regulate both NPS and nutritional status, which reveals their common potential neural regulatory basis. These insights also suggest that treating some sub-symptoms could be used as an approach in lowering the risk of malnutrition, which should not simply be restricted to alleviating NPS. Future studies are required to elucidate the shared mechanisms between the sub-symptoms of NPS and nutrition, and clinical trials are necessary to identify the best strategies to prevent and acutely treat malnutrition in patients with NPS.

## Author contributions

Research design, J.J., H.S., and A.W.; research conduct and original draft preparation, J.J.; data analysis, J.J., A.W. and X.Z.; data collection, J.J., T.J., W.L., L.W., S.J., Y.Z., M.S., and X.Z.; figure preparation, W.L., S.J.; and manuscript revision and fund support, J.X. All authors reviewed the manuscript for important intellectual content and read and approved the final manuscript. The corresponding author attests that all listed authors meet the authorship criteria and that none of the authors who meet the criteria have been omitted. J.X. was the guarantor of this work and, therefore, had full access to all the data in the study and takes responsibility for the integrity of the data and accuracy of the data analysis.

## Funding

This research was funded by the National Key Research and Development Program of China (grant numbers 2021YFC2500100 and2021YFC2500103) and the National Natural Science Foundation of China (grant numbers 82071187 and81870821).

## Ethics approval

This study was conducted in accordance with the Declaration of Helsinki and approved by the Institutional Review Board of Capital Medical University, Beijing Tiantan Hospital (approval number: KY-2021-028-01).

## Informed consent statement

In this study, informed consent was obtained from all participants or their legal guardian.

## Data availability statement

The datasets generated and/or analyzed during this study are available upon request from the corresponding author.

## Conflicts of interest

All authors declare no conflict of interest.
