# Supplementary material for: Clinical and neuroimaging association between neuropsychiatric symptoms and nutritional status across the Alzheimer's disease continuum: a longitudinal cohort study
Source: J Nutr Health Aging. 2024 Feb 9;28(3):100182. doi: 10.1016/j.jnha.2024.100182 (PMC12880567; doi:10.1016/j.jnha.2024.100182)
Supplement: Supplementary file 1 [file mmc1.docx]

**Supplementary table 1 The association of 19 ROIs with nutritional status and general or specific NPS at baseline**

|  | Nutritional statues | | | General NPS | | Psychotic symptoms | |
| --- | --- | --- | --- | --- | --- | --- | --- |
| variables | | OR (95%CI) | *P* values | OR (95%CI) | *P* values | OR (95%CI) | *P* values |
| Sex | | 1.422 (0.805–2.512) | 0.225 | 0.935 (0.495–1.764) | 0.835 | 0.943 (0.449–1.983) | 0.877 |
| Onset of age | | 1.037 (1.001–1.075) | 0.044 | 0.967 (0.929–1.007) | 0.100 | 1.033 (0.986–1.083) | 0.175 |
| left insula | | 0.980 (0.943–1.018) | 0.301 | 0.961 (0.922–1.002) | 0.062 | 0.957 (0.908–1.009) | 0.102 |
| right insula | | 0.996 (0.959–1.034) | 0.835 | 0.990 (0.948–1.033) | 0.636 | 1.024 (0.975–1.075) | 0.339 |
| left amygdala | | 1.001 (0.970–1.032) | 0.961 | 1.001 (0.964–1.038) | 0.977 | 0.976 (0.937–1.016) | 0.236 |
| right amygdala | | 0.997 (0.966–1.028) | 0.832 | 0.989 (0.954–1.026) | 0.564 | 0.973 (0.937–1.011) | 0.163 |
| left caudate nucleus | | 0.997 (0.970–1.024) | 0.833 | 1.004 (0.974–1.036) | 0.776 | 1.004 (0.968–1.041) | 0.831 |
| right caudate nucleus | | 0.987 (0.959–1.016) | 0.386 | 0.972 (0.940–1.005) | 0.094 | 0.964 (0.929–1.002) | 0.061 |
| left putamen | | 1.041 (1.001–1.083) | 0.043 | 1.061 (1.014–1.110) | 0.011 | 1.021 (0.974–1.071) | 0.388 |
| right putamen | | 1.013 (0.969–1.058) | 0.567 | 1.058 (1.003–1.116) | 0.037 | 1.078 (1.020–1.140) | 0.008 |
| left NAc | | 0.990 (0.967–1.013) | 0.382 | 0.971 (0.947–0.997) | 0.027 | 0.980 (0.949–1.011) | 0.195 |
| right NAc | | 0.996 (0.972–1.020) | 0.730 | 1.022 (0.994–1.051) | 0.124 | 1.009 (0.977–1.027) | 0.596 |
| left VTA | | 1.001 (0.988–1.014) | 0.890 | 0.999 (0.984–1.014) | 0.911 | 1.010 (0.993–1.027) | 0.253 |
| right VTA | | 0.996 (0.983–1.009) | 0.554 | 0.994 (0.979–1.008) | 0.404 | 1.002 (0.985–1.018) | 0.830 |
| left LC | | 1.005 (0.994–1.017) | 0.358 | 0.999 (0.986–1.012) | 0.860 | 0.994 (0.979–1.010) | 0.475 |
| right LC | | 0.989 (0.977–1.002) | 0.110 | 0.997 (0.984–1.011) | 0.710 | 1.000 (0.983–1.016) | 0.975 |
| dorsal raphe nucleus | | 1.005 (0.992–1.019) | 0.430 | 1.009 (0.993–1.024) | 0.282 | 1.012 (0.995–1.030) | 0.164 |
| left ACC | | 0.989 (0.962–1.017) | 0.428 | 0.970 (0.939–1.002) | 0.067 | 0.972 (0.939–1.007) | 0.120 |
| right ACC | | 1.010 (0.986–1.035) | 0.399 | 1.009 (0.981–1.037) | 0.545 | 1.025 (0.995–1.057) | 0.108 |
| left hypothalamus | | 1.004 (0.983–1.025) | 0.709 | 1.015 (0.991–1.039) | 0.214 | 0.975 (0.946–1.004) | 0.087 |
| right hypothalamus | | 1.012 (0.993–1.030) | 0.228 | 1.012 (0.990–1.034) | 0.285 | 1.017 (0.993–1.042) | 0.168 |
| constant | | 0.042 | 0.020 | 12.959 | 0.094 | 0.025 | 0.042 |

Continued on Next Page

Continued

|  | Affective symptoms | | Hyperactivity | | Appetite/eating disorders | |
| --- | --- | --- | --- | --- | --- | --- |
| variables | OR (95%CI) | *P* values | OR (95%CI) | *P* values | OR (95%CI) | *P* values |
| Sex | 1.292 (0.717–2.330) | 0.394 | 0.606 (0.343–1.070) | 0.084 | 1.552 (0.656–3.672) | 0.318 |
| Onset of age | 0.970 (0.934–1.007) | 0.109 | 0.974 (0.940–1.010) | 0.157 | 1.044 (0.991–1.100) | 0.108 |
| left insula | 0.946 (0.909–0.985) | 0.006 | 0.945 (0.908–0.984) | 0.005 | 0.987 (0.932–1.045) | 0.645 |
| right insula | 0.983 (0.944–1.022) | 0.386 | 1.030 (0.991–1.071) | 0.132 | 1.019 (0.963–1.078) | 0.516 |
| left amygdala | 1.005 (0.973–1.038) | 0.761 | 0.993 (0.963–1.025) | 0.683 | 0.987 (0.945–1.031) | 0.552 |
| right amygdala | 0.973 (0.941–1.005) | 0.101 | 0.984 (0.953–1.016) | 0.335 | 1.007 (0.961–1.055) | 0.761 |
| left caudate nucleus | 1.009 (0.981–1.037) | 0.545 | 1.005 (0.978–1.033) | 0.720 | 0.994 (0.954–1.036) | 0.776 |
| right caudate nucleus | 0.966 (0.937–0.996) | 0.027 | 0.982 (0.954–1.011) | 0.224 | 0.976 (0.936–1.018) | 0.257 |
| left putamen | 1.044 (1.002–1.088) | 0.040 | 1.048 (1.007–1.090) | 0.021 | 1.030 (0.976–1.088) | 0.282 |
| right putamen | 1.076 (1.024–1.131) | 0.004 | 1.039 (0.993–1.087) | 0.098 | 0.990 (0.929–1.054) | 0.744 |
| left NAc | 0.979 (0.956–1.003) | 0.082 | 0.995 (0.972–1.018) | 0.640 | 0.993 (0.960–1.028) | 0.709 |
| right NAc | 1.018 (0.993–1.045) | 0.166 | 1.006 (0.982–1.031) | 0.609 | 1.027 (0.993–1.063) | 0.124 |
| left VTA | 0.997 (0.983–1.010) | 0.625 | 0.999 (0.986–1.012) | 0.862 | 0.992 (0.973–1.011) | 0.415 |
| right VTA | 0.992 (0.978–1.005) | 0.231 | 1.000 (0.987–1.014) | 0.961 | 1.002 (0.983–1.020) | 0.864 |
| left LC | 0.999 (0.987–1.012) | 0.923 | 1.002 (0.991–1.014) | 0.703 | 1.010 (0.994–1.027) | 0.234 |
| right LC | 0.999 (0.987–1.012) | 0.939 | 1.003 (0.990–1.016) | 0.646 | 1.003 (0.985–1.020) | 0.767 |
| dorsal raphe nucleus | 1.004 (0.991–1.018) | 0.528 | 1.003 (0.989–1.016) | 0.680 | 1.000 (0.981–1.019) | 0.976 |
| left ACC | 0.977 (0.948–1.006) | 0.122 | 0.964 (0.936–0.993) | 0.014 | 0.981 (0.943–1.020) | 0.340 |
| right ACC | 1.031 (1.005–1.058) | 0.018 | 1.020 (0.996–1.045) | 0.103 | 1.011 (0.977–1.047) | 0.527 |
| left hypothalamus | 1.012 (0.991–1.034) | 0.265 | 0.998 (0.977–1.019) | 0.831 | 0.997 (0.966–1.028) | 0.844 |
| right hypothalamus | 1.000 (0.980–1.019) | 0.979 | 0.999 (0.981–1.018) | 0.945 | 0.999 (0.971–1.027) | 0.923 |
| constant | 9.934 | 0.105 | 3.361 | 0.372 | 0.003 | 0.006 |

Abbreviation: ROIs, regions of interest; NPS, neuropsychiatric symptoms; OR, odds ratio; CI, confidence interval; NAc, nucleus accumbens; VTA, ventral tegmental area; LC, locus coeruleus; ACC, anterior cingulate cortex

**Supplementary Table 2 Baseline clinical characteristics of patients lost and not lost to follow-up**

| Variable | Patients followed up (N=165) | Patients dropped out (N=267) | t/χ2/Z | *p* value |
| --- | --- | --- | --- | --- |
| **Demographics** |  |  |  |  |
| Age [years, median (IQR)] | 67.70±7.87 | 66.74±8.65 | -1.154 | 0.249 |
| Sex (female, %) | 93 (56.36) | 171 (64.04) | 2.532 | 0.112 |
| Education [years, median (IQR)] | 12.00 (9.00, 12.00) | 11.00 (8.00, 12.00) | -0.567 | 0.571 |
| Marital status (married, %) | 147 (89.09) | 224 (83.90) | 2.270 | 0.132 |
| *APOEε4* carrier (yes, %) | 54 (32.73) | 89 (33.33) | 0.017 | 0.897 |
| **Medical Histories** |  |  |  |  |
| Hypertension (yes, %) | 71 (43.03） | 121 (45.32） | 0.216 | 0.642 |
| Diabetes mellitus (yes, %) | 26 (15.76) | 56 (20.97) | 1.804 | 0.179 |
| Cerebrovascular disease (yes, %) | 29 (17.58) | 50 (18.73) | 0.090 | 0.764 |
| Coronary heart disease (yes, %) | 32 (19.39) | 54 (20.22) | 0.044 | 0.834 |
| Dyslipidemia (yes, %) | 74 (44.85) | 111 (41.57) | 0.447 | 0.504 |
| **NPS and sub-symptoms** |  |  |  |  |
| NPI [score, median (IQR)] | 5.00 (1.00, 14.50) | 4.00 (0.00, 13.00) | -1.599 | 0.110 |
| NPS (yes, %) | 132 (80.00) | 194 (72.66) | 2.968 | 0.085 |
| Psychotic symptoms (yes, %) | 45 (27.27) | 58 (21.72) | 1.730 | 0.188 |
| Affective symptoms (yes, %) | 113 (68.48) | 159 (59.55) | 3.491 | 0.062 |
| Hyperactivity (yes, %) | 82 (49.70) | 114 (42.70) | 2.016 | 0.156 |
| Appetite or eating disorders (yes, %) | 26 (15.76) | 35 (13.11) | 0.590 | 0.442 |
| Euphoria (yes, %) | 14 (8.48) | 16 (5.99) | 0.980 | 0.322 |
| Sleep and nighttime behavior (yes, %) | 53 (32.12) | 70 (26.22) | 1.745 | 0.186 |
| **Neuropsychological battery** |  |  |  |  |
| MMSE [score, median (IQR)] | 24.00 (17.00, 27.00) | 23.00 (15.00, 26.00) | -1.867 | 0.062 |
| MoCA [score, median (IQR)] | 19.00 (12.00, 22.50) | 17.00 (9.00, 22.00) | -1.759 | 0.079 |
| MNA [score, median (IQR)] | 24.50 (21.13, 26.00) | 24.00 (21.50, 26.00) | -0.863 | 0.388 |
| Nutritional status (well-nourished, %) | 95 (57.58) | 136 (50.94) | 1.807 | 0.179 |
| ADL [score, median (IQR)] | 21.00 (20.00, 28.00) | 21.00 (20.00, 30.00) | -0.006 | 0.995 |
| PSQI [score, median (IQR)] | 5.00 (2.00, 9.00) | 5.00 (2.00, 7.00) | -1.065 | 0.287 |
| CBI [score, median (IQR)] | 4.00 (0.00, 21.00) | 3.00 (0.00, 20.00) | -0.280 | 0.780 |

χ² tests, independent *t*-tests, and Mann-Whitney U test. Data are shown as the mean ± standard deviation, median (IQR), or n (%).

Abbreviation: *APOEε4*, the apolipoprotein E type epsilon 4; NPI, Neuropsychiatric Inventory; NPS, neuropsychiatric symptoms; MMSE, Mini-Mental State Examination; MoCA, Montreal Cognitive Assessment; MNA, Mini-nutritional Assessment; ADL, Activities of Daily Living; PSQI, Pittsburgh Sleep Quality Index; and CBI, caregiver burden inventory.
